# An empirical study on attitudes toward gambling when sportswashing is involved

**DOI:** 10.3389/fpsyg.2023.1147332

**Published:** 2023-10-17

**Authors:** André Syvertsen, Eilin Kristine Erevik, Elise Constance Fodstad, Lisa-Christine Girard, Puneet Kaur, Joakim Hellumbråten Kristensen, Eirin Kolberg, Rune Aune Mentzoni, Arne Magnus Morken, Dominic Sagoe, Ståle Pallesen

**Affiliations:** ^1^Department of Psychosocial Science, University of Bergen, Bergen, Norway; ^2^Norwegian Competence Center for Gambling and Gaming Research, Bergen, Norway; ^3^Centre for Alcohol and Drug Research (KORFOR), Stavanger University Hospital, Stavanger, Norway; ^4^Department of Special Needs Education, Oslo University, Oslo, Norway; ^5^Center for Crisis Psychology, University of Bergen, Oslo, Norway

**Keywords:** sports, gambling attitudes, problem gambling, corruption, moral

## Abstract

Sportswashing is defined as individuals, groups, companies, or countries/regimes’ involvement in sports to improve their own reputation and/or to distract from or normalize wrongdoing. This cross-sectional survey is the first empirical study on sportswashing in relation to gambling. The sample consisted of United Kingdom residents who reported past 12-month gambling (*N* = 786, 50% women, mean age = 45.6, SD = 15.2). We investigated how many were familiar with sportswashing and their attitudes toward gambling when sportswashing is involved. Exploratory and confirmatory factor analysis (CFA) were conducted on the attitudes scale that was developed for the current study. Multiple regressions were used to examine if individual differences in terms of age, gender, personality, moral foundations, political trust and efficacy, and/or gambling risk were associated with such attitudes. Finally, we examined the percentage of people who avoid gambling on teams/events when sportswashing is involved, including group differences in avoidance and motivations for avoidance according to gambling risk. The results showed that only 32% had heard about sportswashing prior to the survey. CFA indicated that attitudes toward sportswashing and gambling as conceptualized in the scale used in the current study can broadly be categorized into two dimensions: How individuals relate to sportswashing when gambling (“self-factor”) and how individuals think gambling companies and regulators should regulate sportswashing and gambling [an “external-factor,” *p* < 0.001, CFI = 0.0.996, RMSEA = 0.090, 90% CI (0.077, 0.104)]. Multiple regressions indicated that measures of individual differences explained a significant amount of variance in self-oriented (*F* (17, 765) = 7.19, *p* < 0.001, adjusted *R*^2^ = 0.12) and external-oriented (*F* (17, 765) = 8.40, *p* < 0.001. adjusted *R*^2^ = 0.14) attitude toward gambling and sportswashing. Avoidance of betting when sportswashing is involved was reported by 43%. The proportion was lower among those with moderate gambling risk/problem gambling (35%) compared to those with no/low gambling risk (45%). It is concluded that further scale development could help elucidate individual differences in attitudes toward sportswashing and gambling. Sportswashing remains an important social issue, and the present study indicates that this has high relevance for the gambling field.

## Introduction

The use of sports in reputation management has a long history, with sports being used in ways that are considered favorable or deceptive/exploitative. Sportswashing is a relatively new concept that involves acts that are more in line with the latter notion (Boykoff, 2022; Fruh et al., 2022; Skey, 2023). Gambling and sports are now also increasingly interwoven (Newall et al., 2019). For instance, gambling sponsorships have increased over the last decade, advertisements portray sports betting as an integral part of engaging in sports, betting language and opportunities often feature in sports commentaries, and individuals report that they experience an oversaturation in marketing of gambling during sporting events (Thomas et al., 2012; Deans et al., 2017; Lopez-Gonzalez et al., 2018; Lopez-Gonzalez and Griffiths, 2018). Given the connection between gambling and sports demonstrated by empirical work such as the aforementioned studies, it appears likely that some gamblers may now reflect on how they perceive and respond to sportswashing, including the intersection of sportswashing and gambling.

Sportswashing is a developing concept, although recent theoretical work helps elucidate this phenomenon and its position in the landscape of similar concepts. Boykoff (2022) defines sportswashing as “a phenomenon whereby political leaders use sports to appear important or legitimate on the world stage while stoking nationalism and deflecting attention from chronic social problems and human-rights woes on the home front” (p. 342) and presents a typology with two primary factors that lend more precision to sportswashing: Political context and audience. The political context can constitute democratic or authoritarian agents and the audience can be domestic or international. For instance, Boykoff (2022) argues that the Salt Lake City Winter Olympics in 2002, which were the first Olympic games after the 9/11 terrorist attack, served to present the U.S. as a safe and benevolent country which strengthened public support for the “War on Terrorism.” This would be categorized as a case where a democratic actor targets primarily a domestic audience, and could arguably be accepted as a case of sportswashing. The inclusion of democratic actors as possible sportswashing agents is relatively new, as earlier use of the construct has been restricted to authoritarian practice/states (Fruh et al., 2022; Skey, 2023).

Skey (2023) explores sportswashing within the landscape of similar terms which describe actors conducting reputation management or international relations work. Sportswashing bears similarity to other terms where washing is used as a metaphor, such as whitewashing. Whitewashing literally refers to painting over structural defects to conceal them and various acts that share such intentions are communicated in its metaphorical use (Fruh et al., 2022). Skey (2023) argues that distraction or deflection is particularly applicable for sportswashing. For instance, while fully concealing human rights violations is challenging within global and free flow of information, supporting sports teams and sport events presents the state in a favorable light and thus helps in reputation management by introducing positive associations. Terms for using sports in reputation management vary by academic discipline and evaluation. Propaganda is a general and pejorative term within the history discipline describing the use of information and practices for false messaging and concealment. Positively laden general terms include diplomacy (international relations), soft power (politics/international relations), and place branding (marketing). Sportswashing seems as such to represent a more sport-specific and interdisciplinary term.

Sportswashing has mostly been discussed as perpetrated by countries/regimes, although individuals, groups, or companies can be included within the term to adapt for evolving practice and interpretation (Fruh et al., 2022). Such definitional refinement was seen as necessary for ‘greenwashing’ in which practice evolved beyond initial definitions (Skey, 2023). Conceptual limitations to sportswashing also depend on the context where the term is employed. The division of positive and negative use of sports for reputation management might not always be clear-cut in practice–designating something as sportswashing or diplomacy depends partly on interpretation. Further, there appears to be little work considering whether alleged cases of sportswashing have been successful (Boykoff, 2022). However, these limitations are likely less detrimental when studying individuals’ perception of sportswashing and how/whether they believe it should influence their own and others actions. In the present study’s context, it is arguably most pertinent that the individual in question interpret something as sportswashing.

Individuals typically attach feelings and identity to sports teams and engage in fan communities, deriving experiences of pride and relatedness. However, when tied to moral violation, as is the case with sportswashing, the individual may enter a state of dissonance (Festinger, 1962). This can conceivably influence individuals’ decision to view and engage in sports related activities, including gambling such as betting on sports teams or in events implicated in sportswashing. Individuals’ qualms about sports betting might be doubly affected in cases when they gamble through gambling operators that themselves have sponsorship deals with sports teams or events implicated in sportswashing. Sportswashing has recently received increased attention due to Qatar 2022 FIFA World Cup in football. Qatar has faced criticism for its human rights record and treatment of migrant workers, and have been accused of hosting the World Cup for sportswashing purposes (Søyland and Moriconi, 2022). Individuals who are aware of sportswashing must choose if and how they want to respond to it. In terms of resistance, two broad categories of response have been proposed: Ending participation or transforming participation (Fruh et al., 2022). The perhaps most obvious and effective ways to demonstrate resistance against sportswashing might be to refuse to attend/watch sporting events or buy supporter equipment in which sportswashing might be involved in order to reduce the sportswashing agent(s) revenue, and/or to try to convince others from doing so (Fruh et al., 2022). Ending or transforming participation in the context of sportswashing and gambling might be a more indirect way of signaling resistance as only the gambling company, and hence not the sportswashing agent(s), receive revenue based on gambling bets. This can include refusing to gamble on events or teams directly involved in sportswashing, or on one’s favorite team when it competes against another team involved in sportswashing.

Personality can predict political attitudes (Gerber et al., 2011) which suggests it could also predict how individuals perceive and relate to sportswashing. The HEXACO personality model, which is based on modern and cross-cultural analyzes of personality adjectives, is an alternative to the Big Five personality model (Ashton et al., 2004). The HEXACO model includes six personality factors: Honesty-humility (H), Emotionality (E), eXtraversion (X), Agreeableness (A), Conscientiousness (C), and Openness (O). The Honesty-humility differentiates the HEXACO from the Big Five model and appears relevant in the context of sportswashing. This trait reflects a person’s level of sincerity, modesty, and fairness (Ashton et al., 2014). Honesty-humility has been shown to predict valuation of equality and collective well-being (Chirumbolo and Leone, 2010; Lee et al., 2010). Sportswashing is commonly associated with corruption, infringement of human rights, and misleading behavior, so it may be hypothesized that individuals high in honesty-humility will be more attentive and critical of it. Further, those low in honesty-humiliation are more likely to prioritize their own interests (Ashton et al., 2014), thus they might continue to gamble and watch sporting events regardless of events/hosts being implicated in sportswashing. One might also expect openness to be related to attitudes and practices regarding sportswashing and gambling, as being preoccupied with and resisting sportswashing appear to be a rather recent trend, and since individuals with higher openness scores tend to be more open for novel ideas and perspectives (Costa and McCrae, 2008).

Another set of dispositions likely to influence attitudes toward sportswashing concerns moral reasoning. Moral foundation theory posits that individuals vary in terms of the moral intuitions they draw upon when deciding right and wrong (Graham et al., 2011, 2013). Moral foundation theory proposes five foundations: Care/harm which concerns the welfare and absence of suffering in others, fairness/cheating which concerns justice and equality, loyalty/betrayal which concerns commitment to groups, authority/subversion which concerns hierarchy and respect for leaders/institutions, and purity/degradation which concerns sacredness and spirituality. Care/harm and fairness/cheating are collectively termed the individualizing moral foundations as they relate to the protection of individuals’ rights and welfare (Graham et al., 2009). Higher endorsement of individualizing moral foundations predicts endorsement of human rights principles (Stolerman and Lagnado, 2020). Claimed examples of sportswashing are the emir of Qatar owning Paris Saint-Germain FC, Qatar hosting the 2022 FIFA World Cup, and a Saudi Arabian public investment fund owning Newcastle United FC. These are all examples where sports clubs or events have close ties to regimes that have been criticized for human rights violations. Consequently, those who base their moral reasoning on care/harm and fairness/cheating might be more likely to have critical attitudes toward sportswashing. Other moral foundations might also predict attitudes toward sportswashing. For example, sportswashing is argued to be wrong in part because it exploits and corrupts the values inherent in sports, which are taken by some to be sacred (Edgar, 2021; Fruh et al., 2022). As such, individuals with higher endorsement of purity/degradation foundation might be assumed to be more critical of sportswashing than their counterparts.

Political trust (e.g., trust in parliament, politicians, political systems) may also be related to individuals’ attitudes toward gambling when sportswashing is involved. Political trust concerns citizens’ general assessment of whether political actors within the political system behave according to citizens’ expectations, making it a mechanism of political congruence and democratic accountability (Hooghe and Kern, 2015; Okolikj et al., 2022). Political trust is an important predictor of citizens’ attitudes toward the overall political system, which reflects on their political behavior. For example, institutional participation such as turnout in elections, contacting government officials, and party membership is higher among citizens with higher levels of trust in politics (Hooghe and Marien, 2013). However, those who are lower on political trust tend to use other non-institutional channels to express their discontent, usually by signing petitions, boycotting, and/or protesting of participation (Kaase, 1999; Hooghe and Marien, 2013). These political behaviors could translate to sportswashing, where non-institutional activities may emerge, such as resistance or boycotting of sports, teams, or events due to low trust that political systems will address and handle sportswashing (Fruh et al., 2022; Søyland and Moriconi, 2022). This could suggest that critical attitudes toward gambling when sportswashing is involved may be inversely associated with political trust. Another factor in political participation is political efficacy which refers to the sense that political action can have an impact on political processes, which can be divided into internal (individual sense of competence for participation) and external (perceived responsiveness of governments/institutions) types (Esaiasson et al., 2015). Political efficacy has been associated with increased political participation, regardless of type (Oser et al., 2022). This could suggest that political efficacy is positively associated with critical attitudes toward gambling when sportswashing is involved because the individual think that they and/or governments/institutions have the power to counteract it thus making involvement (e.g., through forming critical attitudes) worthwhile.

The individual’s level of gambling risk might affect both their attitudes and behavior toward gambling when sportswashing is involved. While findings are mixed, some studies have found that individuals with problem gambling hold more positive attitudes toward gambling overall (Kristensen et al., 2022). Thus, individuals with problem gambling might prioritize their own gambling opportunities and thus be less critical toward and have lower likelihood of avoiding gambling when sportswashing is involved.

The present study is, to the best of our knowledge, the first to examine the association between gambling and sportswashing. Given the lack of previous empirical studies, the present study employs a broad approach in examining the issue of sportswashing and gambling in relation to several variables that might be relevant. The study was guided by the following research questions (RQs):

RQ1: To what extent are gamblers familiar with sportswashing?

RQ2: To what extent do gamblers report avoiding gambling when sportswashing is involved and what motives are associated with such avoidance?

RQ3: Are attitudes toward gambling when sportswashing is involved associated with personality, moral foundations, political trust or efficacy, and/or level of gambling problems?

RQ4: Is the avoidance of gambling when sportswashing is involved and related motives associated with level of gambling problems?

## Materials and methods

### Participants and procedure

The present study is a cross-sectional survey among individuals in the United Kingdom’s general population. The survey was set up using the online survey tool SurveyXact^1^ and administered online in November 2022 through the recruitment service Prolific (prolific.co), with the aim to provide a nationally representative sample in terms of age, gender, and ethnicity. Recruitment continued until 1,000 participant responses were received. This resulted in a sample of 1,021 persons. In all, 48% were women, and the mean age was 45.9 years (SD = 15.6). Participants were only included in the study if they reported gambling at least once during the previous 12 months and accurately rejected two statements (“the earth is flat” and “the capital of England is Lima”), functioning as attention checks, which were failed by *n* = 1 and *n* = 6, respectively. The resulting final sample comprised 786 participants, which consisted of 50% women and had a mean age of 45.6 (*SD* = 15.2). Respondents were compensated with £5 for their participation (estimated to take no more than 30 min) in the study, a sum which was in line with Prolific’s recommendations for a reasonable/good hourly pay (i.e., currently £9 per hour). The dataset, analysis code, study material, and supplementary material are publicly available at https://osf.io/sw4f8/.

### Measures

#### Demographic information

The participants were asked to provide demographic information on age, gender, education level, employment status, personal income after tax, marital status, childcare responsibilities, and ethnicity. Table 1 provides a summary of sample descriptive information regarding age, gender, and ethnicity. See online supplementary material available at https://osf.io/sw4f8/, for sample descriptive information regarding all demographic variables and response alternatives.

**Table 1:** Participant characteristics (*N* = 786).

Characteristic^1^	Mean (*SD*)/*n* (%)
Age	45.64 (15.19)
Gender	
Men	392 (50.00%)
Women	391 (49.87%)
Other	1 (0.13%)
^a^Ethnicity	
White/Caucasian	591 (75.38%)
Mixed	193 (24.62%)
^b^Problem gambling	
No problem	403 (51.27%)
Low risk	225 (28.63%)
Moderate risk	110 (13.99%)
Problem gambling	48 (6.11%)
Had heard about sportswashing prior to survey	249 (31.58%)
^c,d^Self-oriented attitudes toward sportsbetting and sportswashing	4.21 (1.26)
^c,d^Other-oriented attitudes toward sportsbetting and sportswashing	4.89 (1.31)
^c^Avoids betting when sportswashing is involved	337 (42.88%)
^c^Motivation to avoid: Bad luck	2.58 (1.54)
^c^Motivation to avoid: Sportswashers benefit economically	5.82 (1.27)
^c^Motivation to avoid: Signals approval of sportswashers	6.05 (1.10)
^c^Motivation to avoid: Do not want to watch when sportswashing involved	5.46 (1.41)

^a^Participants could also respond Asian, Black/African/Caribbean, Arab, or Other. ^b^As measured by Problem Gambling Severity Index, where the risk categories represent sum scores 0; 1–2; 3–7, and 8+, respectively. ^c^Ranges from 1 (Completely disagree) to 7 (Completely agree). ^d^Higher scores indicate being more critical of sportswashing and gambling.

#### Gambling and problem gambling

Gambling was defined as “staking money on an event where the outcome is partly or completely determined by chance,” adapted with some simplification from Bolen and Boyd (1968), p. 619. After being presented this definition, participants were asked if they had participated in any type of gambling within the last 12 months. Problem gambling was assessed with the Problem Gambling Severity Index (PGSI; Ferris and Wynne, 2001). The PGSI contains nine items, in which five assess problematic gambling behavior (e.g., “When you gambled, did you go back another day to try to win back the money you lost?”) and four assess negative consequences from gambling (“Has gambling caused you any health problems, including stress or anxiety?”). Each item has four response options ranging from “never” (0) to “almost always” (3). Composite scores are categorized into “no problem” (0), “low risk” (1–2), “moderate risk” (3–7), and “problem gambling” (8+). Cronbach’s alpha of the PGSI was 0.91 in the current study.

#### Sportswashing and gambling

Sportswashing was defined as “individuals, groups, companies, or countries or regimes getting involved in sports (e.g., owners of clubs, sponsors and/or organizers of sport events) as a means to improve their own credibility or reputation and/or to distract from or normalize wrongdoing.” Participants were presented with this definition and then asked if they had ever heard about sportswashing prior to the survey (“yes”/“no”). Participants answering yes were then asked to provide an example and to justify in an open response box why their example constituted sportswashing.

The authors developed 12 items assessing attitudes toward gambling and sportswashing with four items pertaining to the individual, bookmakers/gambling operators, and regulators/government, respectively. Item development was informed by previous literature on sportswashing (Fruh et al., 2022; Søyland and Moriconi, 2022), a well-established scale on general gambling attitudes (Orford et al., 2009), and the authors’ expertise in the gambling field. An example item is “I dislike betting on sporting events where sportswashing is involved.” Items were scored on a 7-point scale ranging from 1 (“completely disagree”) to 7 (“completely agree”). Details on psychometric properties are presented below (see “Results”). Full scale items and distributions of responses are reported in Table 2.

**Table 2:** Attitudes toward gambling when sportswashing is involved (*N* = 786).

Item	Responses: 1 (completely disagree) to 7 (completely agree)^a^							Mean (SD)
	1 - *n* (%)	2 - *n* (%)	3 - *n* (%)	4 - *n* (%)	5 - *n* (%)	6 - *n* (%)	7 - *n* (%)	
1. “I have no problems betting on sports teams involved in sportswashing” - Reversed	33 (4.2%)	54 (6.9%)	94 (12%)	175 (22%)	153 (19%)	141 (18%)	136 (17%)	4.69 (1.66)
2. “I am less motivated to gamble on sporting events where sportswashing is involved”	29 (3.7%)	53 (6.7%)	93 (12%)	140 (18%)	177 (23%)	153 (19%)	141 (18%)	4.79 (1.65)
3. “I dislike betting on sporting events where sportswashing is involved”	27 (3.4%)	58 (7.4%)	73 (9.3%)	194 (25%)	149 (19%)	138 (18%)	147 (19%)	4.76 (1.64)
4. “I never check if sportswashing may be involved before I place a bet” - Reversed	254 (32%)	186 (24%)	134 (17%)	118 (15%)	42 (5.3%)	27 (3.4%)	25 (3.2%)	2.60 (1.60)
5. “Bookmakers and gambling operators should not offer gambling opportunities when sportswashing is involved”	30 (3.8%)	36 (4.6%)	85 (11%)	182 (23%)	158 (20%)	141 (18%)	154 (20%)	4.83 (1.62)
6. “There is no need for bookmakers and gambling operators to inform gamblers if sportswashing is involved in the betting opportunities offered” - Reversed	31 (3.9%)	67 (8.5%)	113 (14%)	136 (17%)	160 (20%)	145 (18%)	134 (17%)	4.65 (1.70)
7. “Bookmakers and gambling operators should have a clear policy against sportswashing”	16 (2.0%)	22 (2.8%)	53 (6.7%)	176 (22%)	164 (21%)	156 (20%)	199 (25%)	5.18 (1.50)
8. “Bookmakers and gambling operators should not be concerned about sportswashing in terms of the products they offer” - Reversed	25 (3.2%)	54 (6.9%)	105 (13%)	149 (19%)	176 (22%)	132 (17%)	145 (18%)	4.75 (1.64)
9. “Regulatory authorities should make gambling illegal where sportswashing is involved”	33 (4.2%)	41 (5.2%)	93 (12%)	202 (26%)	157 (20%)	114 (15%)	146 (19%)	4.70 (1.63)
10. “Regulatory authorities should not compel bookmakers and gambling operators to inform gamblers if sportswashing is involved in their gambling products” - Reversed	34 (4.3%)	56 (7.1%)	94 (12%)	163 (21%)	173 (22%)	131 (17%)	135 (17%)	4.68 (1.66)
11. “Regulatory authorities should be concerned about sportswashing in terms of gambling”	19 (2.4%)	25 (3.2%)	58 (7.4%)	122 (16%)	205 (26%)	177 (23%)	180 (23%)	5.19 (1.49)
12. “Regulatory authorities should not have any policy against sportswashing” - Reversed	11 (1.4%)	22 (2.8%)	75 (9.5%)	152 (19%)	163 (21%)	173 (22%)	190 (24%)	5.18 (1.49)

^a^Full response options: 1 = “Completely disagree,” 2 = “Mainly disagree,” 3 = “Somewhat disagree,” 4 = “Neither agree nor disagree,” 5 = “Somewhat agree,” 6 = “Mainly agree,” 7 = “Completely agree.

Participants were also asked to indicate whether they generally avoid betting when sportswashing is involved (“yes”/“no”). Those responding “yes” were then asked to rate their agreement with four types of reasons/motivations for avoiding betting when sportswashing was involved (i.e., wanting to avoid: bad luck, that the sportswashing agents would benefit economically, signaling support, and/or watching the event in which sportswashing is involved; see online supplementary material for more details, https://osf.io/sw4f8/). Items were scored on a 7-point scale ranging from 1 (“completely disagree”) to 7 (“completely agree”). Additionally, participants could supply other reasons in an open-response box (optional).

#### Personality

The HEXACO personality model was assessed with the HEXACO-60 scale which contains 10 questions per personality factor (Ashton and Lee, 2009). Items are scored on a 5-point scale ranging from 1 (“strongly disagree”) to 5 (“strongly agree”). Items were reversed, summed, and averaged so that higher scores indicated higher scores on the personality factors in question. In the present study, Cronbach’s alpha values obtained for each sub-scale were: 0.70 for honesty-humility, 0.67 for emotionality, 0.85 for extraversion, 0.80 for agreeableness, 0.79 for conscientiousness, and 0.79 for openness.

#### Moral foundations

Moral foundations were assessed with the 20-item Moral Foundations Questionnaire (MFQ; Graham et al., 2011). The 20-item MFQ has four items that are summed and averaged for each of the five moral foundations (i.e., care/harm, fairness/cheating, loyalty/betrayal, authority/subversion, and purity/degradation). Items consists of foundation-related concerns and are scored on a 6-point scale ranging from 1 (“not at all relevant”) to 6 (“extremely relevant”) or from 1 (“strongly disagree”) to 6 (“strongly agree”) depending on the question. In the present study, the following Cronbach alpha values obtained for each sub-scale were: 0.65 for care/harm, 0.59 for fairness/cheating, 0.63 for loyalty/betrayal, 0.68 for authority/subversion, and 0.74 for purity/degradation.

#### Trust in political institutions and political efficacy

Four questions on trust in political institutions were taken from the European Social Survey.^2^ Participants were asked to rate on a scale from 0 (“no trust at all”) to 10 (“complete trust”) how much they personally trust the House of Commons and the House of Lords, the United Kingdom legal system, the UK police, and UK politicians. These items were summed and averaged to form an index variable of general political trust (Hooghe, 2011; Hooghe and Kern, 2015). Cronbach’s alpha for the composite score was 0.87 in the present study.

Two questions on external political efficacy were taken from the Comparative Study of Electoral Systems.^3^ Participants were asked to rate whether it makes any difference who is in power and if it makes any differences who people vote for. Ratings were given on a scale from 1 (“it does not make any difference/will not make a difference”) to 5 (“it makes a big difference). These questions refer to external political efficacy, i.e., primarily reflecting the perceived responsiveness of government/political institutions (Karp and Banducci, 2008).

### Ethics statement

The study was exempt from ethical approval based on the Norwegian Centre for Research Data’s guidelines for anonymous surveys. All participants provided informed consent at the start of the survey.

### Data analysis

Statistical analyzes were conducted with R version 4.2.2 (R Core Team, 2022) and exploratory factor analysis with FACTOR version 12.03.02 (Ferrando and Lorenzo-Seva, 2017). We computed distributions of age, gender, gambling risk status, number of individuals who were familiar with sportswashing, number of individuals who avoid sportsbetting when sportswashing is involved, reported motivation for such avoidance, and the distribution of index variables for attitudes toward sportswashing and gambling (see below). Text data from participants on sportswashing examples was organized using the tidytext approach, which enables a word frequency report (Silge and Robinson, 2016, 2017). This included treating common two-word phrases as a unit (e.g., “world cup”), combining near identical and/or common misspelling of words (e.g., “Qatar” and “Quatar”), and removing meaningless stop-words (e.g., “accordingly” and “also”) based on the SMART lexicon (Lewis et al., 2004).

The 12 items assessing attitudes toward gambling and sportswashing were evaluated psychometrically with exploratory factor analysis (EFA) and confirmatory factor analysis (CFA). We divided the sample that had gambled during the previous 12 months (*N* = 786) into two equally sized sub-groups. EFA was conducted on one sub-group (*n* = 393) with a minimum rank factor analysis (MRFA) based on the polychoric correlation matrix (Shapiro and Ten Berge, 2002; Baglin, 2014). We examined eigenvalues and ran parallel analysis (PA) based on MFRA for factor extraction, the latter allows for comparisons of observed versus random explained common variance. In case of more than one factor, oblique rotation was opted for as factors were assumed to be associated with each other. CFA was conducted on the second sub-group (*n* = 393) with the R package *lavaan* version 0.6–12 (Rosseel, 2012) based on weighted least squares means and variances (WLSMV). WLSMV was chosen as it is designed for ordinal level data (Li, 2016). Model fit was evaluated with chi-square (*χ^2^*), the comparative fit index (CFI) and the root mean square error of approximation (RMSEA).

Results from the EFA and CFA (see “Results” for details) informed two index variables reflecting attitudes toward sportswashing and gambling: One capturing how the individual relates to sportswashing when gambling (“self-factor”) and one capturing how the individual thinks gambling companies/regulators should regulate sportswashing and gambling (“external-factor”). We computed Cronbach’s alpha reliability (Cronbach, 1951), average variance extracted (AVE; Fornell and Larcker, 1981), and composite reliability (Bagozzi, 1981) for these index variables. The index variables also served as dependent variables in two multiple linear regressions. Index variables were calculated as mean scores of items included in the factor, so that higher scores reflected being more critical of sportswashing in gambling and with scores ranging from 1 to 7 (0 to 6 in multiple regressions). Each multiple regression included the same independent variables, age, gender, the six personality factors, the five moral foundations, political trust, and moderate risk/problem gambling (versus no problem/low risk). The category of ‘other gender’ was treated as missing in this analysis due to the low number (*n* = 1). Statistical assumptions were met for both models regarding linearity of residuals, multicollinearity, multivariate outliers, and heteroscedasticity. We followed Ferguson’s (2009) benchmarks for interpreting effect sizes. For adjusted *R*^2^, 0.04 constitutes a practically significant effect, 0.25 a moderate effect, and 0.64 a strong effect. For standardized beta, β, 0.20 constitutes a practically significant effect, 0.50 a moderate effect, and 0.80 a strong effect, respectively.

Finally, we compared the proportion of individuals who avoided gambling when sportswashing is involved between those with no problem/low risk and those with moderate risk/problem gambling using Pearson’s Chi-square and calculating Cramer’s V as an effect size. When avoidance was reported, we also compared differences in motivations to avoid by using Wilcoxon rank sum test and Pearson’s *r* as an effect size. Strength of association was assessed with V and *r*, in which 0.20 constitutes a practically significant effect, 0.50 a moderate effect, and 0.80 a strong effect (Ferguson, 2009).

## Results

**Figure 1:**
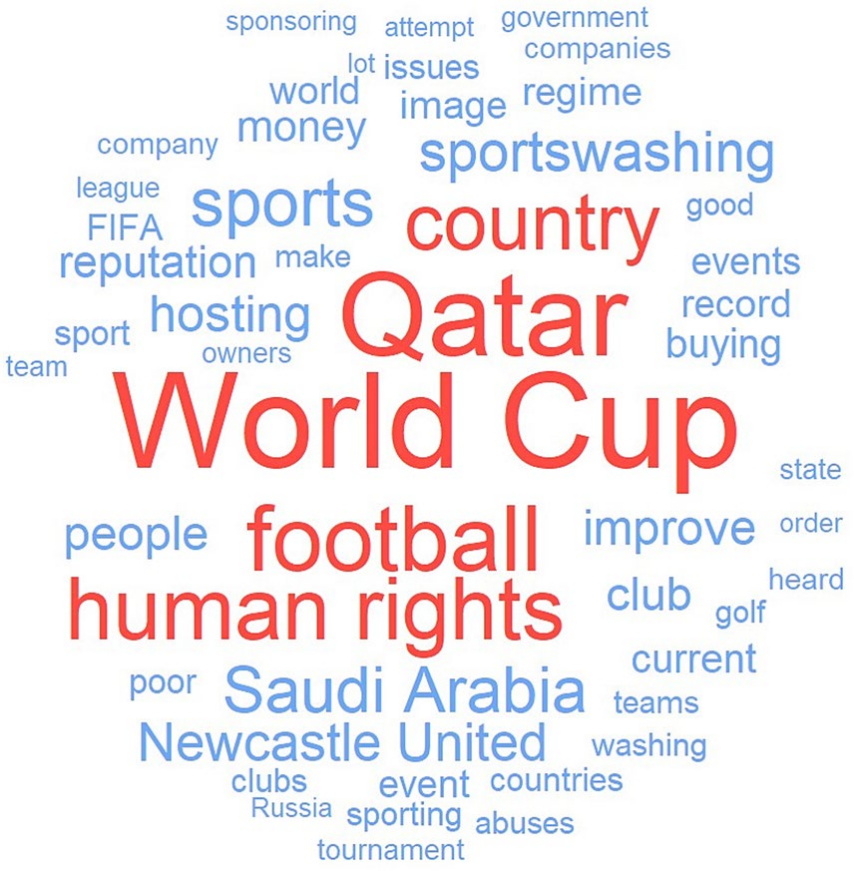
Word cloud showing most frequent words used in examples of sportswashing (five most frequent in red).

Table 1 shows the distribution of age, gender, gambling risk status, and information on key sportswashing and gambling variables. In total 32% of the participants reported having heard about sportswashing prior to the survey and 43% reported that they avoided betting when sportswashing is involved. Regarding motivations to avoid such betting, the strongest motivation, endorsements (ranging from 1 to 7), were for avoiding “signaling approval of sportswashers” (*M* = 6.05), followed by concerns about “sportswashers benefitting economically” (*M* = 5.82), because individuals “do not want to watch when sportswashing is involved” (*M* = 5.46), and finally to avoid “bad luck” when gambling (*M* = 2.58). The latter was the only motivation that respondents tended to disagree with on average. Text analysis of sportswashing examples revealed that World Cup (*n* = 171), Qatar (*n* = 146), football (*n* = 110), and human rights (*n* = 103) were mentioned most frequently. It should be noted that the survey was conducted during the opening week of the 2022 FIFA football World Cup, hosted by Qatar, and frequencies of mentioned terms should be interpreted in light of this. See Figure 1 for word cloud of 50 most frequent words.

### Psychometric evaluation and distribution of attitudes toward sportswashing and gambling

Results from the EFA indicated that the data were fit for factor analysis with Bartlett’s test (*χ^2^* = 4457.4, *df* = 66, *p* < 0.001) and Kaiser-Meyer-Olkin statistic of 0.90. Mardia’s (1970) test revealed excessive kurtosis (*p* < 0.001), which supports the use of polychoric correlations. Results showed that two factors had eigenvalues above 1, but results from the MRFA-PA recommended a one-factor solution. A one-factor model was then evaluated in with CFA on the other sub-sample, but the results indicated poor model fit to our observed data [*χ*^2^ = 615.359, *df* = 54, *χ*^2^/*df* = 11.396, *p* < 0.001, CFI = 0.985, RMSEA = 0.163, 90% *CI* (0.151, 0.175); Hu and Bentler, 1999]. Following this, we then examined the two-factor solution identified by an EFA (which was supported by Kaiser’s criteria, eigenvalue >1.0). The results from the EFA extracting two factors showed that the items that concerned sportswashing and gambling in relation to the person itself loaded upon one factor (items 1 to 4 in the scale, see Table 2), while items concerning sportswashing and gambling in relation to gambling companies and regulators loaded upon a separate factor (items 5–12 in the scale). See supplementary material available at https://osf.io/sw4f8/, for full details on the FACTOR program specifications and output. The CFA on the two-factor solution showed considerable improvement in fit over the one-factor solution [two-factor: *χ*^2^ = 179.847, *df* = 43, *χ*^2^/*df* = 4.182, *p* < 0.001, CFI = 0.0.996, RMSEA = 0.090, 90% CI (0.077, 0.104)]. RMSEA still indicated somewhat sub-optimal fit, but this was deemed acceptable for our current aim which was to identify a model that made theoretical sense, was parsimonious, and had acceptable correspondence to our data (Kline, 2013). Results on reliability analyzes showed that Cronbach’s alpha was 0.76 for “self-factor” and 0.91 for “external-factor,” AVE was 0.55 for “self-factor” and 0.67 for “external-factor,” and composite reliability was 0.78 for “self-factor” and 0.93 for “external-factor.”

The distribution on the index variables derived from the EFA and CFA is reported in Table 1. The results indicate that participants were critical overall of gambling when sportswashing is involved and that they were more critical in relation to gambling operators and regulators (“external-factor”) compared to themselves (“self-factor”).

### Multiple linear regressions on attitudes toward gambling when sportswashing is involved

Results from multiple linear regressions on attitudes toward gambling when sportswashing is involved are presented in Tables 3, 4. The multiple linear regression on self-oriented attitudes [*F* (17, 765) = 7.19, *p* < 0.001, adjusted *R*^2^ = 0.12] explained an overall statistical and practically significant amount of variance in the dependent variable. Further, the results indicate a statistical and practically significant effect size for honesty-humility, and statistically but not practically significant effect sizes for openness and problem gambling/moderate risk. The multiple linear regression on external-oriented attitudes [*F* (17, 765) = 8.40, *p* < 0.001. adjusted *R*^2^ = 0.14] explained an overall statistical and practically significant amount of variance in the dependent variable. Further, the results indicate statistically but not practically significant effect sizes for age, gender, honesty-humility, care/harm, fairness/cheating, and purity/degradation.

**Table 3:** Attitudes regarding how the individual relates to gambling when sportswashing is involved (“self-factor”).

	Original Model			Standardized Model	
Characteristic	Beta	95% CI	*p*	Beta	95% CI
Age	0.00	0.00, 0.01	0.20	0.05	−0.03, 0.12
Gender	0.15	−0.03, 0.34	0.10	0.06	−0.01, 0.13
Honesty-humility	**0.50**	**0.34, 0.66**	**<0.01**	**0.23**	**0.16, 0.31**
Emotionality	−0.01	−0.19, 0.18	0.93	0.00	−0.08, 0.08
Extraversion	0.05	−0.09, 0.18	0.48	0.03	−0.05, 0.10
Agreeableness	0.08	−0.07, 0.23	0.31	0.04	−0.03, 0.11
Conscientiousness	−0.08	−0.25, 0.08	0.33	−0.04	−0.11, 0.04
Openness	**0.15**	**0.01, 0.28**	**0.04**	**0.08**	**0.00, 0.15**
Care/Harm	0.07	−0.07, 0.21	0.36	0.04	−0.05, 0.13
Fairness/Cheating	0.13	−0.04, 0.29	0.13	0.07	−0.02, 0.15
Loyalty/Betrayal	−0.07	−0.20, 0.05	0.23	−0.06	−0.15, 0.04
Authority/Subversion	0.00	−0.14, 0.13	0.94	0.00	−0.10, 0.10
Purity/Degradation	0.06	−0.04, 0.16	0.25	0.05	−0.04, 0.14
Political trust	−0.04	−0.09, 0.00	0.07	−0.07	−0.15, 0.01
Political efficacy (government)	0.05	−0.04, 0.15	0.27	0.06	−0.04, 0.15
Political efficacy (voting)	0.03	−0.06, 0.13	0.51	0.03	−0.07, 0.13
Problem gambling/moderate risk	**−0.26**	**−0.48, −0.05**	**0.02**	**−0.08**	**−0.15, −0.01**
*R*^2^/Adjusted *R*^2^	0.138/ 0.119				

Reference values: Age (0 = 18 years), gender (0 = men, 1 = women), six personality factors (range 0–4), five moral foundations (range 0–5), political trust (range 0–10), government/voting matters (range 0–4), and moderate risk/problem gambling (0 = no problem/low risk, 1 = moderate risk/problem gambling). Statistically significant coefficients in bold. CI = Confidence Interval.

**Table 4:** Attitudes regarding how the individual thinks gambling companies/regulators should relate to gambling when sportswashing is involved (“external-factor”).

	Original Model			Standardized Model	
Characteristic	Beta	95% CI	*p*	Beta	95% CI
Age	**0.01**	**0.00, 0.01**	**0.02**	**0.09**	**0.01, 0.16**
Gender	**0.44**	**0.25, 0.63**	**<0.01**	**0.17**	**0.09, 0.24**
Honesty-humility	**0.25**	**0.08, 0.41**	**<0.01**	**0.11**	**0.03, 0.18**
Emotionality	−0.07	−0.26, 0.12	0.48	−0.03	−0.11, 0.05
Extraversion	0.06	−0.08, 0.20	0.40	0.03	−0.04, 0.11
Agreeableness	0.10	−0.06, 0.25	0.23	0.04	−0.03, 0.12
Conscientiousness	−0.10	−0.27, 0.07	0.25	−0.04	−0.11, 0.03
Openness	0.02	−0.12, 0.16	0.80	0.01	−0.06, 0.08
Care/Harm	**0.18**	**0.03, 0.32**	**0.02**	**0.11**	**0.02, 0.19**
Fairness/Cheating	**0.25**	**0.08, 0.42**	**<0.01**	**0.13**	**0.04, 0.21**
Loyalty/Betrayal	−0.12	−0.25, 0.00	0.05	−0.09	−0.18, 0.00
Authority/Subversion	−0.02	−0.16, 0.11	0.73	−0.02	−0.12, 0.08
Purity/Degradation	**0.18**	**0.07, 0.28**	**<0.01**	**0.15**	**0.06, 0.23**
Political trust	−0.03	−0.08, 0.01	0.16	−0.05	−0.13, 0.02
Political efficacy (government)	−0.03	−0.13, 0.07	0.57	−0.03	−0.12, 0.07
Political efficacy (voting)	0.06	−0.04, 0.16	0.22	0.06	−0.04, 0.16
Problem gambling/moderate risk	−0.05	−0.27, 0.18	0.68	−0.01	−0.08, 0.05
*R*^2^/Adjusted *R^2^*	0.157/ 0.139				

Reference values: Age (0 = 18 years), gender (0 = men, 1 = women), six personality factors (range 0–4), five moral foundations (range 0–5), political trust (range 0–10), government/voting matters (range 0–4), and moderate risk/problem gambling (0 = no problem/low risk, 1 = moderate risk/problem gambling). Statistically significant coefficients in bold. CI = Confidence Interval.

### Group differences in terms of gambling risk/problems in avoiding betting when sportswashing is involved

Results from Pearson’s Chi-square and Wilcoxon rank sum tests comparing group differences in terms of gambling risk/problem in avoiding betting when sportswashing is involved are reported in Table 5. The results show that people with moderate/risk problem gambling are less likely to avoid betting when sportswashing is involved compared to those with no problem/low risk. The difference constitutes 10 percentage points, although the effect is below practical significance (0.20) per effect size benchmark. The results also indicate that there were statistically but not practically significant differences in “motivation to avoid: bad luck” (moderate risk/problem gambler were more likely to endorse), “motivation to avoid: signals approval of sportswashers” (same median, but less variance among no problem/less risk gamblers), and “motivation to avoid: do not want to watch when sportswashing involved (no problem/low risk gamblers were more likely to endorse; Table 5).

**Table 5:** Group differences in avoiding betting when sportswashing is involved and strength of endorsements for related motives.

Characteristic^1^	Moderate and problem, (*n* = 158)	No problem/Low risk, (*n* = 628)	Value of *p*	Effect sizes
Avoids betting when sportswashing involved. *n* (%)	56 (35%)	281 (45%)	0.035	0.072
Motivation to avoid: Bad luck. Md (IQR)	3 (2, 4)	2 (1, 4)	0.014	0.134
Motivation to avoid: Sportswashers benefit economically. Md (IQR)	6 (5, 7)	6 (5, 7)	0.268	0.060
Motivation to avoid: Signals approval of sportswashers. Md (IQR)	6 (5, 7)	6 (6, 7)	0.029	0.119
Motivation to avoid: Do not want to watch when sportswashing involved. Md (IQR)	5 (4, 6)	6 (5, 7)	0.018	0.129

(%) avoiding betting when sportswashing is involved; Md = median, IQR = Interquartile range; 1 = “Completely disagree, 7 = “Completely agree” for motives; Pearson’s Chi-squared test for proportion, Wilcoxon rank sum test comparing motivations to avoid betting when sportswashing is involved (n = 56 versus n = 281 here).

## Discussions

The present study examined the intersection of gambling and sportswashing. An exploratory approach with broad research questions was chosen as this is the first study, to our knowledge, conducted on the topic. RQ1 concerned gamblers’ familiarity with sportswashing. The results indicated that only 32% of the participants had heard about sportswashing prior to the survey, which suggests that this phenomenon is still quite unknown among gamblers in the United Kingdom. The sample distribution on attitudes toward sportswashing and gambling suggested that participants are overall critical toward gambling when sportswashing is involved. Text analysis indicated that the World Cup in Qatar was the most frequent example provided for sportswashing. This is not surprising given that data collection occurred on November 22nd which was at the beginning of the 2022 World Cup. Further, the case of Qatar has been described as a “paradigm case” because the moral violation is regarded as serious and widespread, where the agent is a state, and sports are used in terms of owning clubs or hosting events (Fruh et al., 2022). Examples from other sports mentioned by the participants included Formula 1, golf, and boxing. RQ2 concerned gamblers’ avoidance of gambling when sportswashing is involved. Despite only 32% reporting prior knowledge of sportswashing, 43% of gamblers reported that they would avoid betting when sportswashing is involved. It is likely that some participants report on their intent to avoid such betting in the future, which could be based on them becoming aware of sportswashing through the present survey or that the responding reflect social desirability bias (Chung and Monroe, 2003). Participants mainly agreed (mean ≥ 4, range 1–7) that they avoided such betting because wrong people would benefit economically, it signals support to perpetrators of sportswashing, and because they do not want to watch events where sportswashing is involved. Participants mainly disagreed (mean ≤ 3) that they avoided it because it would give bad luck.

### The role of individual differences in attitudes toward gambling when sportswashing is involved

RQ3 concerned the role of various individual differences in predicting attitudes toward gambling when sportswashing is involved. The overall regression models explained statistically and practically significant amount of variance in how the individual relates to gambling when sportswashing is involved (12% in “self-factor” regression) and how the individual thinks gambling companies/regulators should regulate gambling when sportswashing is involved (14% in “external-factor” regression). However, it is possible that one could predict additional variance by either refining the attitude measure (see “limitations, further research, and conclusions” for discussion on this) and/or with the choice of predictors. Each group of predictors is discussed in turn.

In terms of personality, honesty-humility emerged as a significant predictor in the model on self-oriented attitudes and external-oriented attitudes where this trait was associated with more critical attitudes. This trait was also the strongest predictor overall (*β* = 0.23 in the “self-factor” regression). Individuals high in honesty-humility tend to value equality and collective well-being, which could explain why they would be more critical against sportswashing in relation to gambling because of its connection to moral violation (Chirumbolo and Leone, 2010; Lee et al., 2010). Alternatively, people low in honesty-humility tend to prioritize their own self-interests which could suggest that they are relatively indifferent to sportswashing (and social issues) in general (Ashton et al., 2014) and to gambling where sportswashing is involved specifically. Openness was positively but weakly related to critical attitudes toward gambling where sportswashing is involved regarding the self. Low openness has been associated with higher social conformity orientation which reflects acceptance of conventional norms and authority (Duckitt, 2001; Lee et al., 2010). Hence, those low in openness might thus be less likely to challenge sportswashing and sportswashers (often states/regimes). However, in such a case one might expect that openness should have a stronger association with external-oriented attitudes, which emphasizes the role of regulators/institutions, but no significant association was found here. Another potential explanation to the association between openness and self-oriented critical attitudes toward gambling when sportswashing is involved, might be related to the novelty of resistance toward sportswashing in which individuals with higher openness scores might be early adopters of this trend due to their interest in and openness for new ideas (Costa and McCrae, 2008).

Three moral foundations (i.e., care/harm, fairness/cheating, and purity/degradation) emerged as statistically significant, albeit relatively weak, predictors in the model on external-oriented attitudes toward gambling where sportswashing is involved. Care/harm and fairness/cheating were associated with more critical attitudes. These have collectively been termed the individualizing moral foundations and concern protection of individuals’ rights and welfare, which predict higher human rights endorsement (Graham et al., 2009; Stolerman and Lagnado, 2020). As reflected in the text analysis, participants perceive human rights violation as central to sportswashing. Such violations might resonate more among those higher in individualizing moral foundations, resulting in them being more critical toward gambling when sportswashing is involved. Purity/degradation was also associated with more critical attitudes toward external-oriented attitudes toward gambling when sportswashing is involved. This moral foundation is denoted as a binding moral foundation alongside authority/subversion and loyalty/betrayal. Binding moral foundations have been associated with less support for human rights which could suggest that higher purity/degradation should be associated with less critical attitudes toward gambling when sportswashing is involved (Stolerman and Lagnado, 2020). However, it is plausible that in the current context of sports, those high in this moral foundation react more strongly to sports being used as an instrument and the corruption of the spirit of sports (Edgar, 2021), which could explain why purity/degradation was positively associated with more critical attitudes. There might also be important moderators missing from the current analyzes. For instance, sportswashing can involve specific sports teams (e.g., the case of Newcastle United and Paris Saint-Germain) and stronger activation of moral foundations might take place if the person is a fan of an implicated sports team and/or has stronger interest in sports in general.

The results were not statistically or practically significant regarding the role of political trust and political efficacy in attitudes toward gambling when sportswashing is involved. While this could reflect a lack of association, it is also possible that the present measure did not capture the type of political trust driving an association with attitudes toward gambling when sportswashing is involved. Political trust predicts political involvement, where the direction of attitudes is aimed toward the political institutions of the country, however institutionalized gambling companies’ policies regarding sportswashing may lie outside of this remit. Future studies ought to also measure the association between trust in foreign state actors who own sports teams and are involved in sportswashing. This type of trust may differ from the overall trust in domestic political institutions, which were asked about in the current study. The alleged perpetrator of sportswashing may be acting domestically (United Kingdom in the present study) or internationally, which could affect individuals’ attitudes toward the regulation of sportswashing (including the intersection with gambling). The differentiation between domestic or foreign targets for evaluation could also be relevant for the relationship between political efficacy and attitudes toward gambling when sportswashing is involved. The present measure of political efficacy referred to perceived responsiveness of government/political institutions assessed by whether voting matters or those in power matters, thus referring to domestic political institutions (Karp and Banducci, 2008). However, the text analysis demonstrated that the 2022 Qatar World Cup was the most referenced example of sportswashing which clearly constitutes a foreign case of alleged sportswashing.

Moderate gambling risk/problem gambling was weakly associated with less critical attitudes toward gambling where sportswashing is involved in the “self-model.” Moderate risk/problem gamblers exhibit higher gambling involvement and can experience difficulties abstaining from gambling, difficulties that likely persist even when sportswashing is involved (Potenza et al., 2019). The acknowledgement that one would likely gamble regardless of the presence or absence of sportswashing might lead the individual to experience cognitive dissonance that can be relieved by thinking that one must not care that much about sportswashing as a social issue (Festinger, 1962).

Finally, older age and female gender were associated with more critical attitudes toward gambling when sportswashing is involved in the “external-factor” regression. These demographic groups have been found to have more negative attitudes toward gambling in general, which might explain why they are more negative toward the intersection of gambling and sportswashing as well (Kristensen et al., 2022).

### Avoidance of betting when sportswashing is involved and level of gambling problems

RQ4 concerned the influence of gambling problem/risk in avoidance of gambling when sportswashing is involved. Individuals with moderate gambling risk and problem gambling were less likely to report intent to avoid gambling when sportswashing was involved, compared with individuals with no problem/low risk. This could be due to people with moderate risk/problem gambling experiencing difficulties abstaining from gambling regardless of the presence or absence of sportswashing. In terms of reported motivation for avoidance, those with moderate risk/problem gambling were more likely to report that they avoided gambling when sportswashing was involved because they believed it brought bad luck. This fits well with findings showing that people with problem gambling are more likely to hold irrational beliefs about gambling, including concerning the nature and role of luck in gambling (Toneatto, 1999). The finding also supports the notion that people with problem gambling might base more on their gambling behavior on concerns regarding own gains compared to people without problem gambling.

### Strengths, limitations, further research, and conclusions

The present study has some limitations that should be mentioned. Its cross-sectional design means that we cannot draw conclusions about directionality or causality, and the potential influence of recall and social desirability bias among participants should be considered when interpreting our findings. We used Prolific to recruit participants in the current study and opted for a representative sample in terms of age, gender, and ethnicity. However, our final sample lacked representativeness in terms of individuals with Asian, Black/African/Caribbean, and Arab ethnicity not being represented. In terms of strengths, it should be noted that the risk for demand effects and inattentiveness during responding is low among respondents recruited through Prolific (Peer et al., 2017; Palan and Schitter, 2018). Attentiveness during responding was also confirmed by the very low number of respondents that failed the two attention checks in the full sample (0.1 and 0.6%, respectively). Further, the present study was strengthened by including several psychological constructs that could plausibly be related to attitudes toward gambling when sportswashing is involved such as personality traits, morality foundations, demographic variables, political trust and efficacy, and level of gambling problems. However, greater nuance could have been added to the results by additional gambling measures such as gambling type and gambling frequency. For instance, it is likely that sports bettors show stronger attitudes toward gambling when sportswashing is involved compared to lottery players. Similarly, higher gambling frequency may also predict stronger attitudes. Interpretation of the present findings should also consider that the majority (68%) of participants had not heard of sportswashing prior to the study, as such, their attitudes and/or motivation to avoid sportswashing reflect their evaluation of a novel concept and their future intent. However, it is possible that some of these participants were familiar with the acts described in the definition provided, albeit not the term itself.

Further investigation should be done on assessment and scale development of attitudes toward gambling when sportswashing is involved. The two-factor solution that was obtained in the present study was supported by Kaiser’s criterion (eigenvalue >1.0), although this approach is known for the risk of overestimating factors to retain (Ruscio and Roche, 2012). The “self-factor” was based on 4 items only and it is possible that a more stable two-factor solution would emerge if additional items were developed to capture this factor. Future studies should also investigate if general interest in sports or being a fan of a sports team that has been implicated in sportswashing can impact an individual’s attitudes toward gambling when sportswashing is involved. In terms of the relationship between such attitudes and moral foundations, it is possible that this would be better examined with the help of vignettes given that most participants were unfamiliar with sportswashing (Clifford et al., 2015). Moral foundations such as care/harm moral reasoning (e.g., reading about someone causing harm to an animal) and purity/degradation moral reasoning (e.g., seeing someone desecrating a bible) are typically triggered by specific cases/examples (Graham et al., 2013). Hence, it is possible that individuals need to be exposed to more specific situations for their intuitive ethics to activate.

The present study constitutes, to our knowledge, the first empirical study of gambling where sportswashing is involved. The results indicate that a minority of gamblers had heard of sportswashing, although they were critical toward it. Results suggest that attitudes toward gambling when sportswashing is involved can broadly be categorized as either capturing how the individual relates to this phenomenon, or how the individual thinks gambling companies and regulators should regulate gambling when sportswashing is involved. Individual differences in personality, moral foundations, gambling risk, age, and gender appear to be associated with attitudes toward gambling when sportswashing is involved. Further scale development could help better elucidate individual differences in attitudes toward gambling when sportswashing is involved. Sportswashing remains an important social issue, and the present study indicates that this has significant relevance to the gambling field as well as gamblers systematically vary in their attitudes toward gambling when sportswashing is involved, including in their intent to avoid gambling when sportswashing is involved.

## Data availability statement

The original contributions presented in the study are publicly available. This data can be found here: https://osf.io/sw4f8/.

## Ethics statement

Ethical approval was not required for the study because of the Norwegian Centre for Research Data’s guidelines for anonymous surveys. The studies were conducted in accordance with the local legislation and institutional requirements. The participants provided their written informed consent to participate in this study.

## Author contributions

AS: conceptualization, methodology, data curation, formal analysis, writing–original draft and revision, and visualization. EE, EF, L-CG, PK, JK, EK, RM, and DS: conceptualization, methodology, and writing–review and editing. AM: conceptualization, methodology, data curation, and writing–review and editing. SP: Conceptualization, methodology, writing–review and editing, data curation, project administration, and funding acquisition. All authors contributed to the article and approved the submitted version.

## Footnotes

^1^ surveyxact.com

^2^ www.europeansocialsurvey.org

^3^ https://cses.org/
